# Paper Money and Disease

**Published:** 1904-04

**Authors:** 


### Paper Money and Disease.

IN ITS March edition the JOURNAL referred to the bill before congress to establish post check currency, and suggested that it receive the support of physicians because it is in the interest of preventive medicine. Our contemporary, that stalwart newspaper the *Buffalo Evening News,* in its issue of March 10, 1904, commented as follows:

FOR CLEAN MONEY.

The BUFFALO MEDICAL JOURNAL is one of the most ably edited publications of its class in this country and we are glad to see that it is taking hold of the question of a cleaner currency and thus sustaining the position which the *News* took on this question years ago, and has maintained ever since.

The BUFFALO MEDICAL JOURNAL says:

> Clean money is an important desideratum as every physician will attest. Soiled currency is a medium for the transmission of disease; this, too, every physician knows. A remedy is offered in congress through a bill to create post check currency. In other words, it is proposed to create a system of bank notes, payable to the order of the individual named on the face of the bill. This can be sent through the mails, properly indorsed, similarly to personal checks or drafts. It is canceled after one payment made through its medium, hence the danger of transmitting disease is reduced to a minimum. Fractional currency could thus be created which would meet a great demand in the payment of small sums to persons at a distance. This proposition being in the interest of the prevention of disease should receive the approval of every physician.

So far so good, but the MEDICAL JOURNAL does not go far enough. It should advocate the calling in by the United States Government of this truthfully-called filthy lucre in shape of bills that are worn with so much service, carrying microbes around from one state to another and spreading disease and death. Of course, it might be asked by unthinking persons why doctors or the BUFFALO MEDICAL JOURNAL should take up a subject which would help to cut off a source of their revenue. The reply is that doctors have enough to do and always will have without epidemics. It is high time some one at Washington brought this currency question before Congress and the BUFFALO MEDICAL JOURNAL can help to bring this about.

The *News* is quite correct; its point is well taken. It is not sufficient for congress to content itself with passing the post check currency bill, without a clause authorising and requiring the immediate redemption of soiled money. Indeed, we are inclined to favor the practice of the Bank of England, in redeeming every note as fast as paid in, soiled or clean; because, no matter how new a bank-note or how clean it looks, if once used it is sure to become contaminated with disease germs.

In support of this statement we cite a Washington despatch to *The Tribune*, under date of March 14, 1904, which reads:

Representative Gaines, of Tennessee, today laid before the House Committee on Banking and Currency a letter from Dr. Thomas Darlington, Health Commissioner of New York, giving the result of his investigation of coin and paper money to determine the extent it may be a conveyer of disease germs.

Bacteria, according to the report, will not live longer than forty-eight hours on silver, nickel and copper coins. Two dirty bills were washed and one was found to contain 135,000 bacteria, the other 126,000. Two comparatively new bills contained respectively 2,250 and 2,000 germs. The report says: "Upon all were found staphylococci, proving that money may carry disease."

We are indebted to the *Buffalo Evening News*, both for its complimentary reference to the JOURNAL and for pointing out the importance of canceling soiled paper money. We did not enter upon a discussion of this phase of the subject in the paragraph quoted by the *News*, because we thought it better to heat one iron at a time, but on reflection, we think the post check currency bill should contain the redemption clause referred to and we will gladly second the efforts of the *News*, so ably made for several years, toward the end sought.

